# Celiac crisis, a rare and profound presentation of celiac disease: a case report

**DOI:** 10.1186/s12876-018-0784-0

**Published:** 2018-05-02

**Authors:** Elizabeth Ann Forrest, Mark Wong, Srinivasa Nama, Siddharth Sharma

**Affiliations:** Diagnostic, Emergency and Medical Services, Gold Coast Hospital and Health Service, Gold Coast University Hospital, 1 Hospital Blvd, Southport, Gold Coast, Queensland 4215 Australia

**Keywords:** Celiac disease, Gluten, Celiac crisis, Subacute degeneration of the spinal cord, Vitamin B12 deficiency, Pulmonary embolism

## Abstract

**Background:**

Celiac crisis is a life-threatening manifestation of celiac disease and is rare in adults, with only a handful of cases documented worldwide and mostly in children.

**Case presentation:**

A profoundly emaciated 43-year-old female presented with profuse diarrhoea, shortness of breath, left leg swelling with ulceration and immobility (Body Mass Index (BMI) = 14.7 kg/m^2^). The patient had normal anion-gap metabolic acidosis (pH = 7.16) with persisting hypokalemia, hyponatremia, hypomagnesemia and hypophosphatemia. In addition, severe vitamin deficiencies and coagulopathy were present. A computed tomography pulmonary angiogram (CT-PA) revealed bilateral massive pulmonary embolism causing infarction, arising from a left lower limb extensive deep vein thrombosis (DVT). Bone marrow suppression was seen on aspirate. The patient developed severe urosepsis in her immunocompromised state. Prolonged lower limb weakness despite supportive therapy, rehabilitation and strict adherence to a gluten-free diet prompted the magnetic resonance imaging (MRI) confirmed diagnosis of subacute combined degeneration of the spinal cord due to Vitamin-B12 deficiency.

**Conclusions:**

Celiac crisis is a rare and potentially life-threatening presentation of celiac disease, often a diagnosis of exclusion. Subacute combined degeneration of the spinal cord should be considered in patients with chronic Vitamin B12 deficiency presenting with neurological symptoms.

## Background

Celiac disease is a systemic immune-mediated enteropathy characterised by injury to the small intestinal mucosa. It is triggered by dietary intake of gluten in genetically susceptible individuals with class II human leukocyte antigen (HLA) DQ2 and DQ8 [1]. With up to 1% of the population diagnosed, clinical manifestations include diarrhoea, abdominal distension and failure to thrive [2]. It occasionally presents in adults as iron deficiency and osteopenia. The gold standard for diagnosis of celiac disease is duodenal biopsy, revealing lymphocytosis, crypt hyperplasia and villous atrophy in active disease [3]. Celiac disease is treated with a strict gluten-free diet. Celiac crisis, is a rarely documented life-threatening presentation of the disease. Mainly presenting in children, celiac crisis causes profuse intractable diarrhoea with severe metabolic disturbances (such as acidosis and hypokalemia), hypotension, neuromuscular weakness, cardiac arrhythmias and respiratory failure. An adult female presenting with celiac crisis is presented in this report.

## Case presentation

A 43-year-old female presented to our facility with profuse diarrhoea, shortness of breath, swollen left leg with ulceration and associated weakness with immobility. This was on a background of 20-kg of unintentional weight loss over a 12-month period. The patient was profoundly emaciated (BMI = 14.7 kg/m^2^), hypotensive (87/60 mmHg) with cool peripheries, had a moderately distended abdomen and a discoloured swollen left leg. Her past medical history was significant for biopsy confirmed celiac disease 3-years earlier.

On admission, the patient had a normal anion-gap metabolic acidosis (pH 7.16, (7.35–7.45)) in the setting of chronic malabsorption with ongoing losses. Profound hypoalbuminemia (< 15 g/L (35–55)) and globalised oedema was noted. Intravenous therapies were used to correct severe electrolyte imbalances including persistent hypokalaemia, hyponatremia, hypomagnesemia and hypophosphatemia. Coagulation profile revealed INR 2.1 (1–2), APTT 45 s (26–39), with thrombocytopenia and anaemia, haemoglobin 78 g/L (120–160). The patient was deficient in iron (Ferritin 19 μg/L (20–200), Transferrin Saturation < 6% (15–45)), Vitamin B12 (164 pmol/L (200–680)), zinc (5 μmol/L (8–18)), copper (5 μmol/L (11–14)) and selenium (0.5 μmol/L (0.7–1.4)). Vitamin B12 and iron deficiencies were treated timely with intramuscular and intravenous replacement, respectively.

Ultrasound of the left leg revealed extensive left lower-limb DVT. CT-PA of the chest revealed bilateral pulmonary embolism, associated pulmonary infarction and two cavitating lung lesions. Provoking factors for her DVT included prolonged pre-hospital immobility and severe hypoalbuminemia. She was subsequently commenced on therapeutic Enoxaparin. Infectious and autoimmune causes including systemic lupus erythematosus, antiphospholipid syndrome, sarcoidosis, tuberculosis, Q-fever, viral hepatitis and human immunodeficiency virus were excluded. Type 1 Diabetes Mellitus was also excluded. A large ulcerative skin lesion developed on the patient’s left dorsum foot. Wound healing was impaired by chronic venous stasis, immunosuppression and peripheral oedema.

The patient developed urosepsis requiring treatment with Piperacillin/Tazobactam, Gentamicin and subsequently Ciprofloxacin. Her blood cultures and urine were positive for *Enterobacter cloacae*. Intravenous 5% Albumin infusions were given for hypovolemia and hypoalbuminemia.

**Fig. 1:**
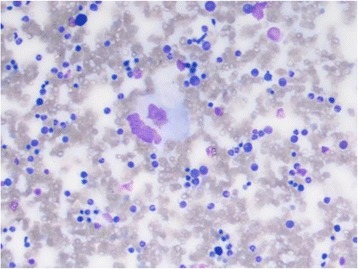
Bone marrow aspirate showing dysplastic features including bi-nuclear megakaryocyte due to severe copper deficiency

**Fig. 2:**
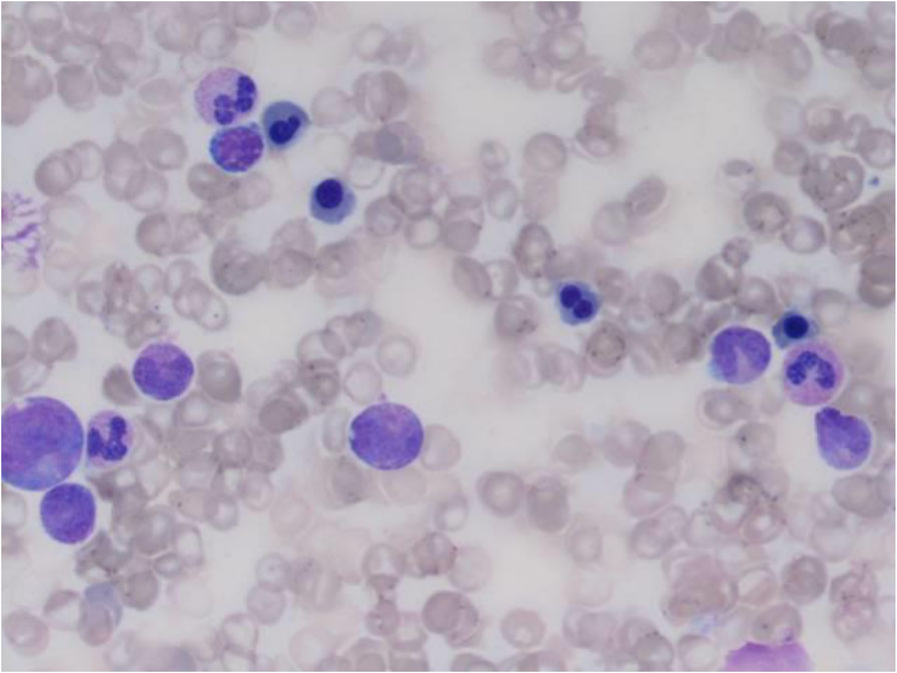
Bone marrow aspirate showing dysplastic features including bi-nuclearity and nuclear lobulation of red blood cells in bone marrow

**Fig. 3:**
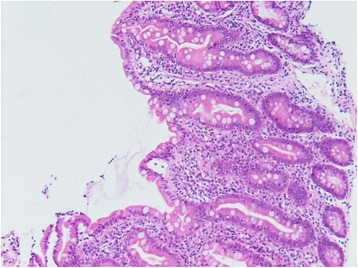
Duodenal biopsy showing villous atrophy, × 20 magnification, stain hematoxylin and eosin

**Fig. 4:**
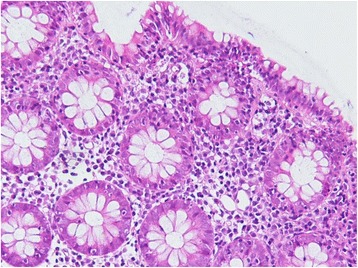
Colonic biopsy showing microscopic feature of lymphocytic colitis, × 40 magnification, stain hematoxylin and eosin

Given the patient’s profound clinical presentation, the treating clinicians had a strong suspicion for malignancy. A positron emission tomography (PET) scan revealed nil evidence of fludeoxyglucose (FDG) avid malignancy. Subsequent bone marrow and trephine aspirate revealed changes related to copper deficiency and severe iron deficiency (Figs. 1 and 2). Once stable, upper and lower endoscopy was performed to investigate for lymphoma. Histological analysis of gastrointestinal biopsies showed villous atrophy and intraepithelial lymphocytosis consistent with severe active celiac disease in the second part of the duodenum and lymphocytic colitis in the colon (Figs. 3 and 4). Duodenal biopsy Modified Marsh classification was 3c, in keeping with symptomatic celiac disease. Anti-tissue transglutaminase was elevated at 608 units. *Helicobacter pylori* infection was excluded as a cause for the patient’s presentation, returning a negative urease (Campylobacter-like organism) test.

The patient was treated with high caloric gluten-free nasogastric and oral feeding regimes, these included elemental and semi-elemental feeds. As the patient was not meeting her target daily oral caloric intake, nasogastric feeds were commenced overnight to assist in weight gain in the context of persistent diarrhoea. A gradual resolution of diarrhoea was noted over a one-month period with a strict gluten-free diet and bridging Loperamide. After comprehensive history taking and investigation, it was concluded that the patient’s celiac crisis was likely triggered by poor dietary compliance and consequent severe malnutrition.

Intensive inpatient rehabilitation was commenced for deconditioning and peripheral neuropathy. Weight gain was recorded over time. Subacute left distal radius and ulnar fractures were discovered, likely from a minor fall one-month prior to admission. Due to the patient’s slow progression in rehabilitation and ongoing lower-limb neurological symptoms a magnetic resonance image (MRI) of the spine was performed. Neurologists diagnosed subacute combined degeneration of the spinal cord, due to chronic Vitamin B12 deficiency. Owing to this diagnosis, the patient continued to be wheelchair-bound and was discharged to a supported care facility. In total, the patient spent 30 days as an acute inpatient and 77 days in inpatient rehabilitation. She is still being followed up by our hospital outpatient haematology, gastroenterology, endocrinology and dietetic service.

## Discussion

Celiac disease is a systemic immune-mediated enteropathy characterised by injury to the intestinal mucosa, affecting mostly the small intestine [1]. It is triggered by dietary intake of gluten in genetically susceptible individuals with class II human leukocyte antigen (HLA) DQ2 and DQ8 [2, 3]. Prevalence of celiac disease is most common in Western countries, with up to 1% of the population diagnosed [1]. Celiac disease rarely presents with life-threatening complications, however there have been a handful of documented cases of celiac crisis, mainly in children [4].

This is a case of severe presentation of celiac disease which was life-threatening. Such a presentation involving haemodynamic instability, electrolyte imbalance, hypoalbuminemia and acidosis has been described as celiac crisis in the literature. A presentation of celiac disease of such severity is rare and thus can be misdiagnosed, particularly when patients have a variety issues. Due to this, it was paramount in this case to rule out other possible causes of her presentation before it was confidently attributed to celiac crisis.

Corticosteroids have been used in other countries as part of the treatment regime of celiac crisis [4, 5]. Steroids were not given to our patient due to her immunocompromised state and co-current sepsis. The aetiology of celiac crisis is unclear; however, it has been hypothesized that stress stimulus such as surgery, infection or pregnancy can be potential triggers [4].

Celiac crisis is life threatening presentation of celiac disease. With a limited amount of published cases in adults, its pathology is rarely diagnosed and is usually a diagnosis of exclusion. With celiac disease affecting a large proportion of the community, clinicians should be aware of the long-term consequences of celiac crisis, the importance of early diagnosis and its prevention. The most common presentation of celiac crisis is profuse diarrhoea, hypotension and muscle weakness [6]. Hypokalaemia is a key feature on blood work-up. Treatment is with supportive therapies and a strict gluten-free diet. Steroids can be considered, but should be used with caution, having been found to exacerbate hypokalemia [7]. Screening for vitamin and mineral deficiencies should be undertaken in patients presenting with coeliac crisis, as the consequences of prolonged deficiencies can cause significant morbidity. This case demonstrates the results of severe Vitamin B12 deficiency due to malabsorption, with the diagnosis of subacute degeneration of the spinal cord during the patient’s rehabilitation journey.

## Abbreviations

APTT: Activated partial thromboplastin time
BMI: Body Mass Index
CT-PA: Computed tomography pulmonary angiogram
DVT: Deep vein thrombosis
FDG: Fludeoxyglucose
HLA: Human leukocyte antigen
INR: International Normalised Ratio
MRI: Magnetic resonance imaging
PET: Positron emission tomography
